# Changes of EEG beta band power and functional connectivity during spaceflight: a retrospective study

**DOI:** 10.1038/s41598-025-96897-5

**Published:** 2025-04-18

**Authors:** Adrián Quivira-Lopesino, María Sevilla-García, Pablo Cuesta, Sandra Pusil, Ricardo Bruña, Patrique Fiedler, Ana Maria Cebolla, Guy Cheron, Michael Funke, Fernando Maestu

**Affiliations:** 1 Center for Cognitive and Computational Neuroscience, Universidad Complutense de Madrid, Madrid, Spain; 2 Department of Pediatrics, McGovern Medical School, The University of Texas Health Science Center at Houston, Houston, TX USA; 3 Department of Radiology, Rehabilitation, and Physiotherapy, School of Medicine, Universidad Complutense de Madrid, Madrid, Spain; 4 Department of Experimental Psychology, Cognitive Processes, and Speech Therapy, School of Psychology, Universidad Complutense de Madrid, Madrid, Spain; 5 Department of Electric Engineering, Universidad Politécnica de Madrid, Madrid, Spain; 6 Health Research Institute of the Hospital Clínico San Carlos (IdISSC), Madrid, Spain; 7 Institute of Biomedical Engineering and Informatics, Technische Universität Ilmenau, Ilmenau, Germany; 8 Laboratory of Neurophysiology and Movement Biomechanics, Université Libre de Bruxelles, Brussels, Belgium

**Keywords:** EEG, Spaceflight, Brain, Beta activity, Neuroscience, Neurology, Medical research

## Abstract

Spaceflight exposes astronauts to unique conditions like microgravity, which may affect brain function, though it remains underexplored compared to other physiological systems. Astronauts often report temporary neurological symptoms, such as disorientation, visual disturbances, and motor issues, potentially linked to structural and electrophysiological brain changes. To investigate this, electroencephalography (EEG) is a reliable tool to study brain activity in space, measuring oscillatory activity and functional connectivity (FC). This study analyzed EEG data from five male astronauts during three stages: pre-flight, during low Earth orbit (LEO), and post-flight in a 2-min task-free eyes-closed (EC) condition followed by another 2-min of eyes-open (EO) condition. The focus was on beta band (12–30 Hz) activity, which is associated with motor control and proprioception. Results showed increased beta power during spaceflight when compared to pre-flight (EC: p < 0.01) and post-flight (EC: p < 0.01; EO:* p* < 0.05) conditions. FC strength also increased during spaceflight when compared to pre-flight (EO: *p* < 0.05) and post-flight (EC: *p* < 0.01; EO: *p* < 0.01) conditions. These differences were found primarily in the sensorimotor cortex (SMC) and frontotemporal regions, suggesting the brain’s adaptation to altered vestibular and proprioceptive inputs during microgravity. As these results reflect astronaut’s movement adaptation to microgravity, this study highlights the importance of understanding central nervous system (CNS) changes during spaceflights to ensure optimal performance and protect astronaut’s health during long-duration missions.

## Introduction

During spaceflights, human’s health and performance is affected by environmental conditions such as isolation, radiation, and microgravity^1,2^. Understanding how such an extreme environment could affect human physiology is crucial to safeguard the well-being of the astronauts and mission success.

Although research in different systems such as cardiovascular, ocular, and others have been frequently investigated, research on the impact of spaceflight on the central nervous system has been scarce. Research on changes in brain functions and structure^3–5^ is of great importance to develop strategies that could mitigate some of the negative effects during exploration class missions^6–8^. While severe impairments have not been documented in astronauts, they have typically reported temporary disorientation, spatial illusions, visual disturbances, sleep alterations, motor deficiencies, and reduced performance^9–11^. These subjective reports seem to be associated with morphological/structural brain changes^12,13^ as well as with electrophysiological changes^14–16^. Surveying the brain’s function during all aspects of missions will help address their relevance and potential clinical significance.

Although the assessment of crewmember’s cognitive function and performance has been primarily dominated by neuropsychological tests^5,14,17,18^, these batteries are subjected to limitations^19^, especially in differentiating subjects in preclinical stages from healthy participants^20^. That is why techniques such as electroencephalography (EEG) and magnetoencephalography (MEG) are effective in identifying preclinical biomarkers and monitoring the progression of brain changes^21^.

Structural changes have been observed in astronaut’s brain MRIs (Magnetic Resonance Imaging) after returning from a 6-months mission aboard the International Space Station (ISS), and it has been estimated that more than 50% of the crewmembers may be affected by such structural changes^12,22^. However, due to the limitations in measuring these changes in space, alternative methods are required. In this context, EEG has proven to be a feasible and reliable neurophysiological technique better suited for studying brain changes before, after, and most importantly during spaceflight^14–16,23–25^. EEG is non-invasive, the recordings can be obtained as many times as needed, the equipment is light and small, and the feasibility for high-quality recordings with dry-EEG electrodes has been already demonstrated^24^.

EEG directly measures global neuronal electrical activity, and it has been used on more than 50 previous flight missions as a part of polysomnography and cognitive studies^26–28^. The dynamics of EEG rhythms can be characterized by power spectrum and synchrony measurements^29^, which can be interpreted as the responsiveness of brain areas and their communication, respectively. Spectral power represents the amount of activity in a specific frequency band [delta (2–4 Hz), theta (4–8 Hz), alpha (8–12 Hz), beta (12–30 Hz), and gamma (30–45 Hz)]. Synchrony can be measured by functional connectivity (FC) analysis, which refers to the synchronization between the measured activity of two or more brain regions in terms of phase or amplitude, indicating statistical dependencies between them^29,30^.

EEG high temporal resolution^31^ allows the detection of both rapid and long shifts of brain activity / responsiveness. Considering this evidence, it becomes vital to broaden our insight into how cognitive processes are influenced by the conditions during short (from a few minutes to a few weeks), and long (from one month to a year or longer) spaceflights. The relevance of utilizing EEG for functional assessment is emphasized by the fact that modifications in brain function could manifest before structural brain abnormalities^32^. The reliability of this method has been proven in clinical research^33^.

When in low Earth orbit (LEO), astronauts are in a weightlessness condition. During this time, astronauts need to adapt to the new environment, where the microgravity causes altered vestibular and proprioceptive inputs that lead to a sensorimotor adaptation^6,12,34^. Because of this, upon return to Earth, the astronauts exposed to long-duration missions need to readapt to the gravitational environment. Several astronauts have reported disturbances in perception, spatial orientation, posture, walking patterns, and eye-head coordination^35,36^.

One of the EEG frequency bands typically associated with sensorimotor activity is the beta band. The beta band activity is prevalent in the sensorimotor cortex (SMC), both in the precentral gyrus and postcentral gyrus^37^, and it is related to proprioceptive processing, motor activity, and motor learning^37–39^. These oscillations increase during stable postures and movement cancelation, and decrease during movement planning and execution^40^. This cortical beta activity has been associated with an increased interneuron-mediated GABA activity^41,42^ and acts as a top-down inhibitory rhythm^40^.

The analyzed dataset used in this study is a part of the NEUROSPAT experiment^15,16^. We performed a retrospective analysis of the EEG data obtained during long-duration ISS missions, to evaluate functional brain characteristics in three different conditions: pre-flight (ground level), in LEO, and post-flight (ground level). Our objective is to assess the changes of power and FC in the beta band, and the persistence of these changes after returning to Earth. Due to the relationship between beta frequency band and motor control, we hypothesize that microgravity conditions could modify the brain activity in this frequency band.

## Methods

The dataset was collected as part of the NEUROSPAT study (AO-2004, 118)^10,15,16,43^, which involved five male astronauts with an average age of 54.2 ± 2.6 years. These participants spent approximately six months (174.6 ± 19.9 days) in low Earth orbit. To standardize sleep duration before the recordings, astronauts completed a sleep survey and were given 8.5 h of rest the preceding night. The recordings were not carried out within 48 h after air travel that crossed more than 4 time zones, nor after work shifts leading to a time shift greater than 4 h, nor the day following imposed sleep deprivation, nor following demanding mental or physical activities, such as centrifuge training, vestibular countermeasures tests, and extravehicular activities. Astronauts were instructed to maintain their usual caffeine intake and to abstain from alcohol or medication for 16 h before the recordings. Recordings were conducted at approximately the same time each day, with a margin of ± 2 h, preferably in the morning. Participants were assessed in three conditions (Table 1).

**Table 1:** Average EEG recording times of all 5 astronauts: pre-flight (second column), in-flight (third column), and post-flight (fourth column).

	Pre-flight (on earth)	In-flight (aboard ISS)	Post-flight (on earth)
Time (days)	66.8 ± 9.0	8.8 ± 1.8	3.0 ± 0.4
	42.6 ± 0.9	54.6 ± 3.7	7.0 ± 1.2
	28.0 ± 0.4		16.8 ± 0.64
			20.2 ± 1.04

*Data are means and standard deviations.

### Ethics statement

All experimental procedures were approved by the European Space Agency Medical Care Committee and the NASA Johnson Space Center Institutional Review Board for Human Testing, in compliance with the Helsinki Declaration of 1964. Each participant provided written informed consent before the experiment began.

### Data acquisition

EEG was used to measure brain activity in all participants during two-minute, task-free eyes-closed (EC) and eyes-open (EO) conditions^15^. On Earth, participants were comfortably seated in a chair, while in space, they were free-floating with their movement restricted by a waist belt attached to straps and secured to metal rings on the racks of the Columbus module in the ISS. To avoid visual distractions, astronauts wore a face mask within a cylindrical tube attached to the laptop screen. This setup was identical for recordings on Earth and in the ISS.

EEG data were collected at a sampling rate of 1116 Hz using the 59-channel electroencephalogram mapping module (MEEMM) of the European physiology module. This system was installed in the Columbus module of the ISS, and recordings were made at the European Astronaut Center (Köln, Germany) or in Star City (Moscow). The MEEMM employed a dedicated physical reference electrode located on the right earlobe. Some post-flight sessions at the Johnson Space Center (Houston) used an asalab 64-channel amplifier (ANT Neuro BV, Hengelo, Netherlands) in a standard lab environment with a 1024 Hz sampling rate. This amplifier is a stationary DC-EEG system with a common average reference. Electrode impedance on the scalp was maintained below 5 kΩ throughout all sessions.

### Data preprocessing

A set of identical 55 channels, standardized according to the 10-10 system, was selected from both the MEEMM and asalab systems to ensure a homogeneous layout and spatial coverage of the head. Bad channels were automatically detected by assessing the mean power spectral density (PSD) in the 70–100 Hz frequency range. A channel was marked as bad if its PSD exceeded the mean PSD + three standard deviations across all 55 channels in the dataset^24^. These identified bad channels were then interpolated using spherical splines^44^, and the DC offset of each channel was removed. After bad channel correction, data were re-referenced to a common average reference. Ocular artifacts were identified and removed using principal component analysis (PCA) in ASA software (ANT Neuro BV, Hengelo, Netherlands), with components removed if they accounted for 95% of the variance within the noise subspace. Muscle artifacts were automatically detected and excluded using the FieldTrip package^45^. Any remaining artifacts were removed following expert visual inspection. The resulting clean data was divided into four-second epochs with a two-second overlap, yielding an average of 23 ± 3 epochs for eyes-closed (EC) and 18 ± 4 epochs for eyes-open (EO) across subjects and conditions. Finally, for the power and functional connectivity analysis, these clean EEG time series were band-pass filtered between 2 and 45 Hz. This filtering used a high-order (1500) finite impulse response (FIR) filter with a Hamming window and was executed using a two-pass filtering process with 2 s padding. We therefore applied it to avoid any signal characteristics changes due to filter-related phase shifts.

### Source reconstruction

Source activity was estimated using a template MRI based on the New York Head (ICBM-NY)^46^, with a 3-layer BEM head model, a regular volumetric grid with a 10 mm spacing source model, and default electrode positions. The forward model was solved using OpenMEEG^47^. For each subject, sources were independently reconstructed using the exact low-resolution brain electromagnetic tomography (eLORETA) method^48^ with a regularization factor of 10⁻⁸. Each source location was assigned a label based on the 90 regions defined in the Automated Anatomical Labeling (AAL) atlas^49^.The whole brain anatomical model here used comprised 78 cortical and subcortical areas mapped by the AAL atlas (excluding the cerebellum, basal ganglia, thalamus, and olfactory cortices), resulting in a total of 1202 source positions for subsequent analysis.

The power spectrum for each source was computed for each trial using the averaged perdiodogram approach with multi-taper Discrete Prolate Spheroidal Sequences (DPSS) with 1 Hz smoothing. Relative power was calculated by normalizing the power spectrum at each source position to the total power across the 2–45 Hz range. The average power for the anatomical model within the beta frequency band was determined by averaging across all epochs and relevant sources, and summing the corresponding frequency steps. This resulted in a reconstructed power matrix for each condition, organized by stages x participants.

To quantify FC, we employed the phase locking value (PLV) metric^50^. PLV is a robust measure of phase synchronization that assesses the consistency of phase differences between the time series of two brain source positions. The reliability of this metric has been substantiated by its high inter-session stability^51^. For the anatomical model, symmetrical matrices of 1202 × 1202 sources were obtained by averaging PLV values across trials for each condition and participant. Then, these matrices were averaged to obtain one single nodal strength value (also known as weighted global connectivity), that represented a marker of whole brain FC, for each participant and condition.

### Statistical analysis

Statistical analysis was conducted using Prism 10 Software (GraphPad version 10.0.2 https://www.graphpad.com/, San Diego, CA, USA). The power spectrum and functional connectivity (FC) values obtained under the three conditions were statistically compared. Differences in beta band relative power and functional connectivity (FC) strength were also assessed between the eyes-closed and eyes-open conditions. Depending on the number of independent variables, data normality (Shapiro–Wilk test), and equality of variances (sphericity test) of the groups, either a one-way ANOVA or a two-way repeated measures ANOVA with Tukey’s multiple comparison test was applied. For repeated measures with reduced sphericity, Geisser and Greenhouse’s correction method was used. Results are presented as mean ± standard deviation (SD), with *p* values noted as follows: *p < 0.05, **p < 0.01, ***p < 0.001. Additionally, q values with a significance level > 5 corresponds with a minimum *p* value of 0.05.

## Results

### Changes in beta band relative power

**Fig. 1:**
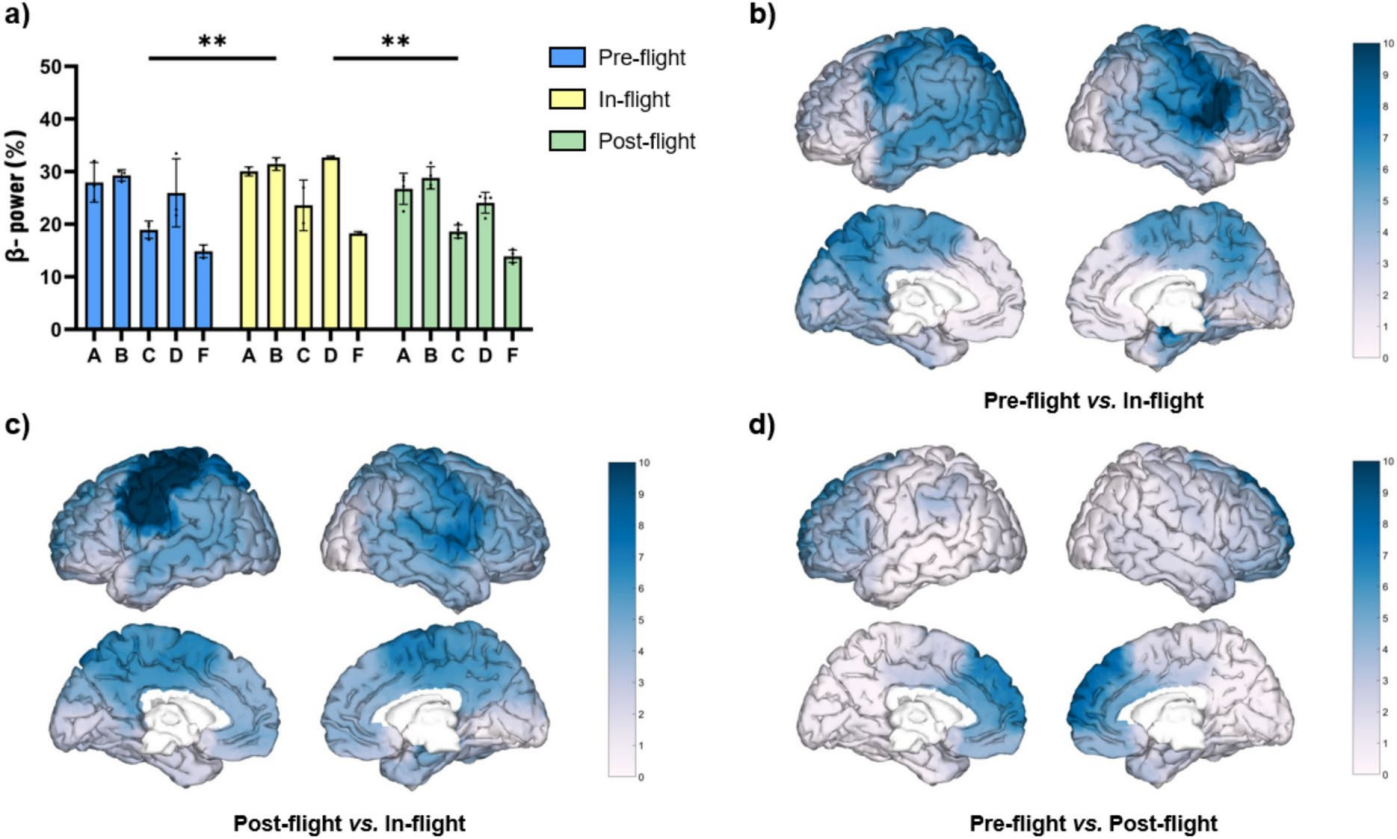
Changes in beta band relative power (eyes-closed) between flight conditions. (**a**) Statistical comparison between conditions. The bar graph depicts the mean ± SD of the beta band power for each flight condition (*p < 0.05, **p < 0.01, ***p < 0.001). (**b**–**d**) Brain figures in the dashed boxes represent the areas with higher statistical power changes in the beta band comparing areas between (**b**) pre-flight versus in-flight conditions, (**c**) post-flight versus in-flight conditions, (**d**) pre-flight versus post-flight conditions. The colorbar is displayed as a family-wise corrected significance level of q value > 5, corresponding with a minimum *p* value of 0.05. The q statistic value was obtained from the results of the post-hoc Tuckey test of the multiple comparison corrections. Thus, the darker the blue color represents brain regions with higher statistical power. The five subjects are mentioned by the respective code letter under each bar. Brain figures were generated using ‘Image Processing Toolbox’ and ‘Medical Imaging Toolbox’ from Matlab R2023b (version 23.2, https://www.mathworks.com/products/matlab.html).

All individual subjects showed an increase of beta band relative power during eyes-closed (EC) under the in-flight condition (aboard ISS station) (Fig. 1). As a cohort, the beta band power was found to be significantly increased (F = 11.92, p < 0.001) during the in-flight condition when compared to the pre-flight (p < 0.01) and post-flight conditions (p < 0.01) (Fig. 1a). These changes were observed across different areas (Fig. 1b–d). Right hippocampus, right Rolandic operculum, and right inferior frontal gyrus (opercular), showed the most considerable differences in beta band power (higher q value) during the in-flight condition compared to the pre-flight condition (Fig. 1b). Left precentral gyrus and left postcentral gyrus showed the most significant differences in beta band power during the in-flight condition compared to the post-flight condition (Fig. 1c). Although no statistically significant overall differences were observed between the pre-flight and post-flight conditions, the medial right superior frontal gyrus showed the most considerable differences (Fig. 1d).

**Fig. 2:**
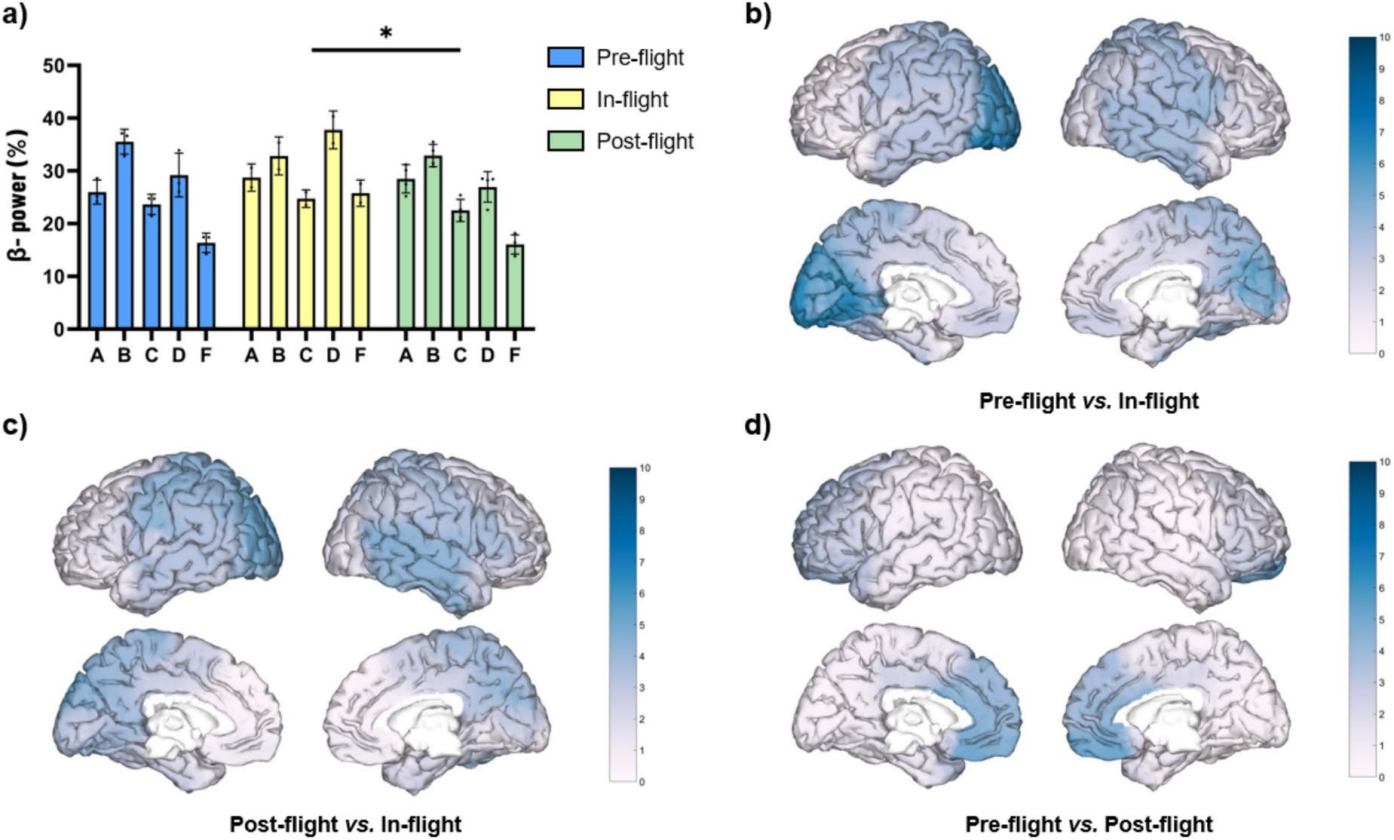
Changes in beta band relative power (eyes-open) between flight conditions. (**a**) Statistical comparison between conditions. The bar graph depicts the mean ± SD of the beta band power for each flight condition (*p < 0.05, **p < 0.01, ***p < 0.001). (**b**–**d**) Brain figures in the dashed boxes represent the areas with higher statistical power changes in the beta band comparing areas between (**b**) pre-flight versus in-flight conditions, (**c**) post-flight versus in-flight conditions, (**d**) pre-flight versus post-flight conditions. The colorbar is displayed as a family-wise corrected significance level of q value > 5, corresponding with a minimum *p* value of 0.05. The q statistic value was obtained from the results of the post-hoc Tuckey test of the multiple comparison corrections. Thus, the darker the blue color represents brain regions with higher statistical power. The five subjects are mentioned by the respective code letter under each bar. Brain figures were generated using ‘Image Processing Toolbox’ and ‘Medical Imaging Toolbox’ from Matlab R2023b (version 23.2, https://www.mathworks.com/products/matlab.html).

Additionally, we evaluated the beta band relative power during eyes-open (EO) and found that not all subjects showed an increase of beta band power under the in-flight conditions (Fig. 2) when compared to the pre-flight or post-flight conditions. As a cohort, the beta band power was found to be significantly increased (F = 11.15, *p* < 0.001) during the in-flight condition when compared to the post-flight condition (*p* < 0.05) (Fig. 2a). These slightly significant changes were observed in the left cuneus and the left superior occipital lobe (Fig. 2c). No statistically significant overall differences were observed during the in-flight condition when compared to the pre-flight condition, or in the pre-flight versus post-flight comparison (Fig. 2a). However, at the area level, the pre-flight condition showed lower beta band power when compared to the in-flight condition principally in the occipital lobe (Fig. 2b). For the pre-flight versus post-flight comparison, the pre-flight condition showed a slightly higher beta band power in the frontal gyrus (Fig. 2d).

**Fig. 3:**
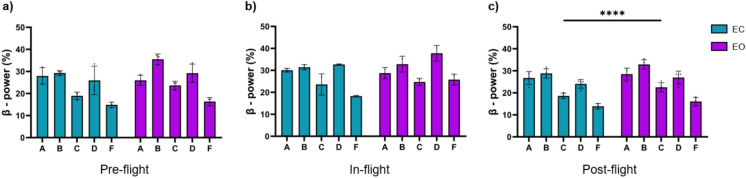
Differences between eyes-closed and eyes-open in beta band relative power between flight conditions. (**a**) Comparison between eyes-closed and eyes-open in the pre-flight condition (*p* = 0.1184). (**b**) Comparison between eyes-closed and eyes-open in the in-flight condition (*p* = 0.3495). (**c**) Comparison between eyes-closed and eyes-open in the post-flight condition (*p* < 0.0001). Bar graphs depict the mean ± SD of the beta band power for each flight condition per subject. The dark blue in a bar indicates the eyes-closed condition, whereas the light blue bar indicates eyes-open condition. The five subjects are mentioned by their respective code letter under each bar. (*p < 0.05, **p < 0.01, ***p < 0.001).

There were beta band power differences between eyes-closed and eyes-open conditions (Fig. 3). Higher relative power was found during eyes-open in the post-flight condition (*p* < 0.0001) (Fig. 3c).

### Changes in beta band FC strength

**Fig. 4:**
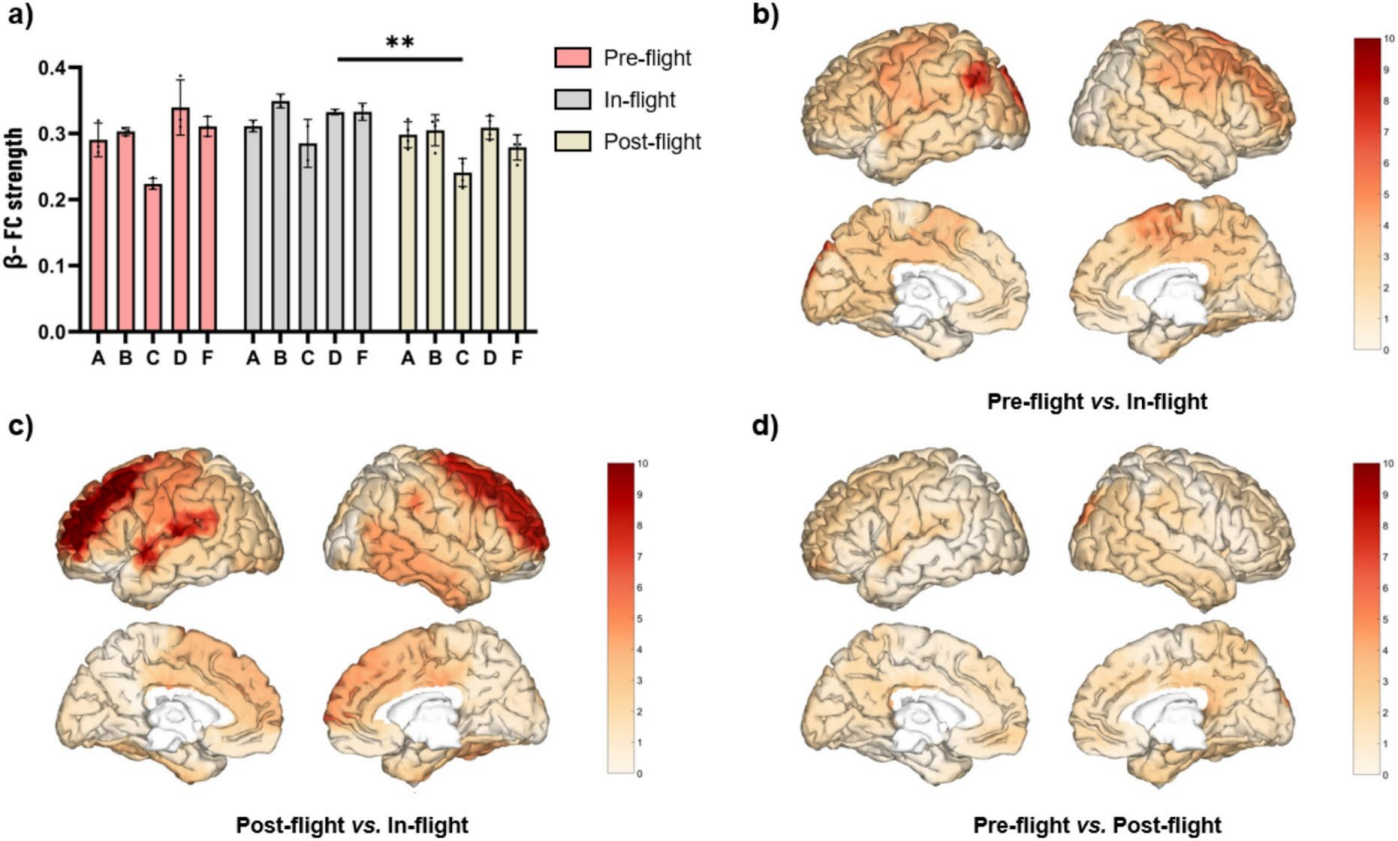
Changes in beta FC strength (eyes-closed) between flight conditions. (**a**) Statistical comparison between conditions. The bar graph depicts the mean ± SD of the beta band FC strength for each flight condition (*p < 0.05, **p < 0.01, ***p < 0.001). (**b**–**d**) Brain figures in the dashed boxes represent the areas with higher statistical FC changes in the beta band comparing areas between (**b**) pre-flight versus in-flight conditions, (**c**) post-flight versus in-flight conditions, (**d**) pre-flight versus post-flight conditions. The colorbar is displayed as a family-wise corrected significance level of q value > 5, corresponding with a minimum *p* value of 0.05. The q statistic value was obtained from the results of the post-hoc Tuckey test of the multiple comparison corrections. Thus, the darker the red color represents brain regions with higher statistical power. The five subjects are mentioned by the respective code letter under each bar. Brain figures were generated using ‘Image Processing Toolbox’ and ‘Medical Imaging Toolbox’ from Matlab R2023b (version 23.2, https://www.mathworks.com/products/matlab.html).

As a cohort, beta band eyes-closed (EC) FC strength significantly increased (F = 9.908, *p* < 0.001) during the in-flight condition when compared to the post-flight (*p* < 0.01) condition (Fig. 4a). These changes were observed across different areas. Significant changes in beta band FC strength were mainly observed in the prefrontal cortex, showing the most significant differences (highest q values), and in the superior temporal gyrus (Fig. 4c). No significant differences were observed between pre-flight and either in-flight (Fig. 4b) or post-flight (Fig. 4d).

**Fig. 5:**
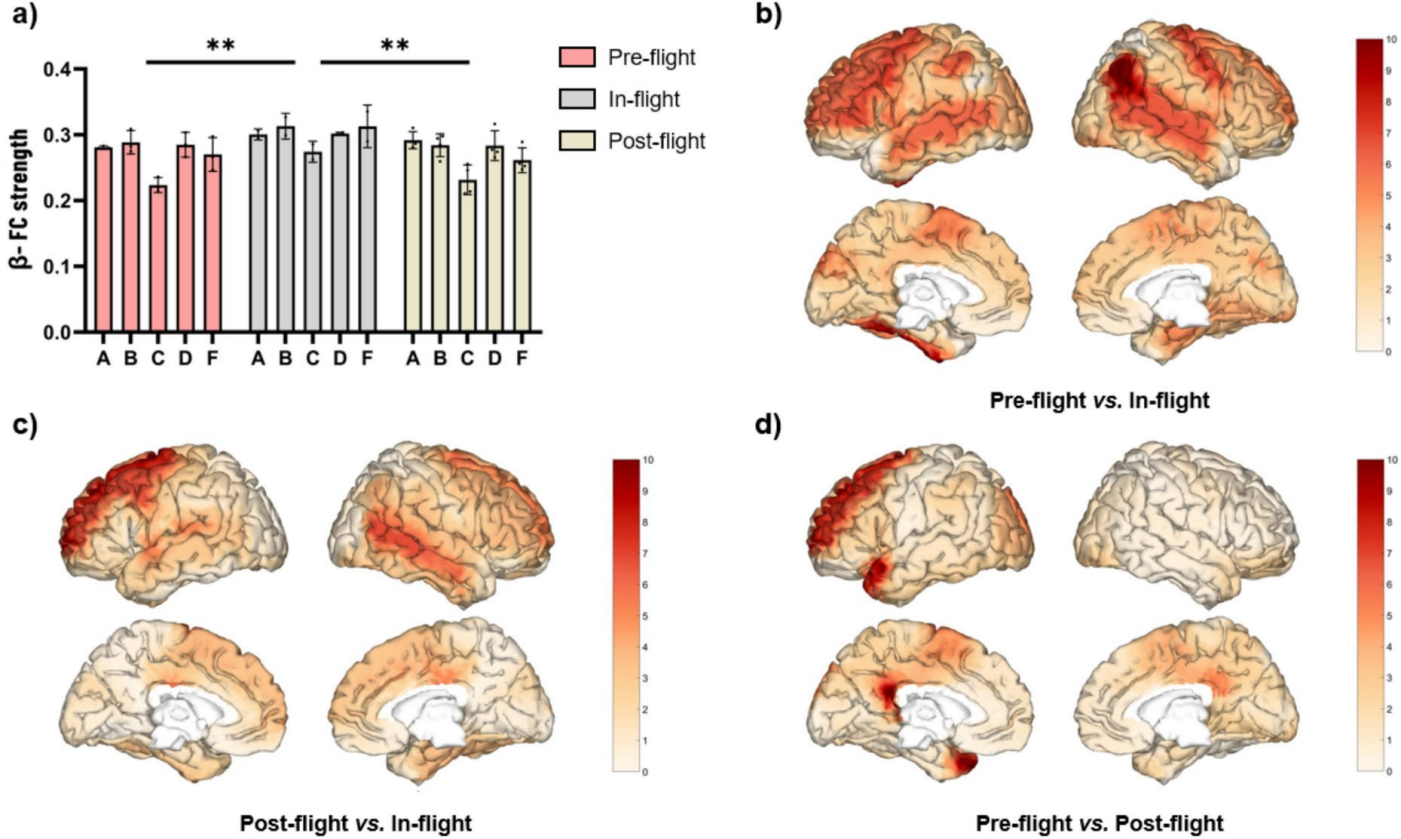
Changes in beta FC strength (eyes-open) between flight conditions. (**a**) Statistical comparison between conditions. The bar graph depicts the mean ± SD of the beta band FC strength for each flight condition (*p < 0.05, **p < 0.01, ***p < 0.001). (**b**–**d**) Brain figures in the dashed boxes represent the areas with higher statistical FC changes in the beta band comparing areas between (**b**) pre-flight versus in-flight conditions, (c) post-flight versus in-flight conditions, (**d**) pre-flight versus post-flight conditions. The colorbar is displayed as a family-wise corrected significance level of q value > 5, corresponding with a minimum *p* value of 0.05. The q statistic value was obtained from the results of the post-hoc Tuckey test of the multiple comparison corrections. Thus, the darker the red color represents brain regions with higher statistical power. The five subjects are mentioned by the respective code letter under each bar. Brain figures were generated using ‘Image Processing Toolbox’ and ‘Medical Imaging Toolbox’ from Matlab R2023b (version 23.2, https://www.mathworks.com/products/matlab.html).

Additionally, we evaluated the beta band FC strength during eyes-open (EO) and found an increase of FC strength (F = 10.62, *p* < 0.001) in all subjects under in-flight conditions compared to pre-flight (*p* < 0.05) and post-flight (*p* < 0.01) conditions (Fig. 5a). These changes were observed across different areas (Fig. 5b–d). Left Rolandic operculum, right angular gyrus, and bilateral precentral gyrus showed the most considerable differences in beta band FC (higher q value) during the in-flight condition compared to the pre-flight condition (Fig. 5b). Left superior frontal gyrus showed the most significant differences in beta band FC during the in-flight condition compared to the post-flight condition (Fig. 5c). Although no statistically significant overall differences were observed between the pre-flight and post-flight conditions, the superior temporal gyrus and the posterior part of the cingulate gyrus showed the most considerable differences (Fig. 5d).

**Fig. 6:**
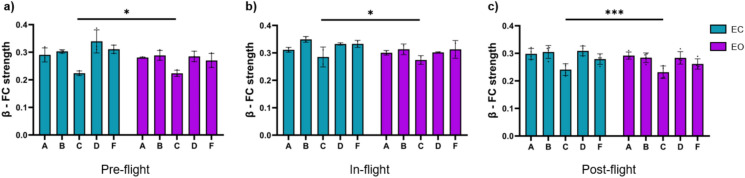
Differences between eyes-closed and eyes-open in beta band FC strength between flight conditions. (**a**) Comparison between eyes-closed and eyes-open in the pre-flight condition (*p* < 0.05). (**b**) Comparison between eyes-closed and eyes-open in the in-flight condition (*p* < 0.05). (**c**) Comparison between eyes-closed and eyes-open in the post-flight condition (*p* < 0.001). Bar graphs depict the mean ± SD of the beta band power for each flight condition per subject. The dark blue in a bar indicates the eyes-closed condition, whereas the light blue bar indicates eyes-open condition. The five subjects are mentioned by their respective code letter under each bar (*p < 0.05, **p < 0.01, ***p < 0.001).

There were beta band FC strength differences between eyes-closed and eyes-open conditions (Fig. 6). Higher FC strength was found during eyes-closed in all flight conditions (Fig. 6a–c) (pre-flight: *p* < 0.05; in-flight: *p* < 0.05, post-flight: *p* < 0.001).

## Discussion

In our research we have assessed electrophysiological changes, measured with whole brain EEG before, during, and after a spaceflight. We have shown that power and FC signatures are altered by the different environments that astronauts endure during a spaceflight. In particular, our results point to an increase in beta power and FC during the time in space that appears to recover when astronauts return home. Monitoring these alterations emerges as an important milestone in understanding how spaceflight can affect brain function.

Spaceflight plays a crucial role in advancing our understanding of the universe and pushing the boundaries of human capability. In recent years, there have been significant advancements in this field, notably with private companies joining traditional space agencies like NASA and ESA. The unique environment of space, including microgravity, radiation exposure, and isolation, poses significant challenges to human health^1,2^. Understanding these health risks helps scientists develop countermeasures, such as exercise routines, nutrition plans, and medical interventions, to maintain astronauts’ well-being. Investigating the health of astronauts in space is also vital for ensuring the safety and success of long-duration missions.

Previous studies have already identified anatomical^22,34^, electrophysiological^15,23^, and physical^35,52^ modifications after long-duration spaceflight missions. However, the implications of these changes are still unclear. Proprioception, widely known to be partially localized in the SMC^53^, plays a key role in the fine coordination of gross and fine motor actions by integrating multiple sensory inputs, including vision, touch, and vestibular functions^54^. In microgravity, all these inputs experience significant modifications, leading to alterations in proprioception-related mechanisms such as: lower limb strength, gaze stabilization, balance, or eye-head coordination, among others^6,36,52^. Due to these alterations, astronauts need to adjust to a new model of movement by depending more on visual cues^55^.

These changes in the sensory inputs would be reflected as electrophysiological and/or anatomical modifications in the brain. The beta frequency activity is commonly linked with motor control^39^ and proprioceptive processing^56^, and has been localized in the SMC^37^. Our study findings indicate that long-term missions can produce related neurophysiological changes, as evidenced through EEG-derived whole brain analyzes.

Compared to pre- and post-flight conditions, the SMC showed a significant increase in beta band power during the flight, especially on the left side, in the five subjects (Fig. 1). These variations across different flight stages may be attributed to adaptations to the microgravity environment. However, as beta activity is associated with posture stabilization and movement cancellation^40^, another potential explanation for these results is the difference in participants’ postural tone during EEG recordings; they were seated during the pre- and post-conditions, whereas in space they were floating, secured only by a belt around their waist. Additional research is necessary to clarify the cause of these results.

Regarding the FC strength results (Fig. 4), the largest increases in beta band were localized in areas linked with highly important cognitive functions during weightlessness in space: the superior frontal gyrus is involved in spatial working memory^57^, the medial frontal gyrus in performance monitoring^58^, the superior temporal gyrus in visual search and spatial perception^59^, and the angular gyrus in spatial processing, specifically in the verticality assessment^60^. The altered proprioceptive integration would trigger the reorganization of motor control strategies, requiring new plans and the development of new motor schemes. This would induce a reorganization of local primary motor regions as well as brain areas involved in motor control and motor schemes evidenced in the increased FC over the prefrontal regions.

The available data from this study alone leaves some unanswered questions. We have to consider that in these extreme conditions during spaceflight, other factors than microgravity could be influencing the beta band activity (isolation, radiation, mood disorders). Although our results could be partially influenced by these factors, the specificity of the brain changes over the primary motor cortex as well as over the motor control regions lead us to link these EEG results to the effects of microgravity. In addition, we excluded the possibility that these changes were driven by arousal or excitation levels, as previous studies have reported opposite results to our findings^7,61^. Another important question is the timing and progression of changes during spaceflight. This cannot be reliably inferred from just two in-flight recordings used in our analysis. Moreover, the dataset had a very limited number of participants, and gender balance was lacking in both datasets, with no female participants included despite known gender differences in neurophysiological data^62,63^. Furthermore, the lack of cognitive, structural, cardiovascular, or physiological markers that would be useful to establish correlations, make it difficult to interpret some of these results. One limitation is the lack of both electrode positions and anatomical information (MRIs) of the astronauts at any point during spaceflight, making it necessary the use of a template to estimate the source activity. As a result, the errors introduced by brain shifts occurring in microgravity will be smaller than those arising from variations in electrode placement between sessions. Subsequent studies with a larger cohort and these additional markers to measure are crucial to elucidate the clinical and operational significance of these changes. Definitively, new studies simulating microgravity on earth should confirm these findings. However, it is crucial to continue acquiring more data during spaceflights to extend the interpretability of these results.

## Data Availability

All relevant data will be available from the Laboratory of Neurophysiology and Movement Biomechanics at Université Libre de Bruxelles (acebolla@ulb.ac.be) upon request and after approval from the European Space Agency Medical Board (ESA-MB) and the NASA Johnson Space Centre Institutional Review Board (NASA-IRB). Authors do not own the data, but others can request data from ESA and NASA. The authors confirm that they did not have any special access or request privileges that others would not have.
